# Is there a “European Paediatrics”?

**DOI:** 10.3389/fped.2024.1326157

**Published:** 2024-04-25

**Authors:** Hans Jürgen Dornbusch, Ronald Kurz, Stefano del Torso, Adamos Hadjipanayis, Alfred Tenore

**Affiliations:** ^1^ Medical University of Graz, Graz, Austria; ^2^ University Hospital for Paediatrics and Adolescent Medicine, Medical University Graz, Graz, Austria; ^3^ ChildCare WorldWide, CCWWItalia OdV, Padova, Italy; ^4^ Medical School, European University Cyprus, Nicosia, Cyprus; ^5^ California University of Science and Medicine, Colton, CA, United States

**Keywords:** children, adolescents, health care organizations, research, education, social welfare

## Abstract

The journey from birth to adulthood is paved with threats to health and wellbeing, rendering this age group with its invaluable future potential particularly vulnerable. Therefore, children and adolescents deserve medical attention of the highest professional level based on solid, well founded training guidelines, the availability of a well-coordinated platform for the continuous acquisition of knowledge, exchange of ideas, and collaboration on research and clinical projects, and comprehensive continuing education. For the European region these crucial specifications are met to varying degrees by three major paediatric organisations: the European Academy of Paediatrics (EAP) with the European Board of Paediatrics (EBP) as the paediatric section of the European Union of Medical Specialists (UEMS PS), the European Paediatric Association (EPA/UNEPSA) and the European Confederation of Primary Care Paediatricians (ECPCP). A major goal of this paper is to call for the closest possible collaboration between these organizations in advocating for the health and rights of European children and adolescents and in effectively fostering the paediatric profession with a strong, unified voice.

## Introduction

Children and adolescents, who make up almost 20% of the European population, present particular health issues and frequently unmet needs. Comprehensive care of this vulnerable age group requires specific expertise and continuous advocacy. Over the last sixty years, three major European organisations have been created that have dedicated their activities to supporting these goals. The oldest of the three is the European Academy of Paediatrics (EAP), founded in 1961 as the “Confederation of European Specialists in Paediatrics” (CESP). The EAP aims to advocate, promote, and set standards for achieving the highest quality of care for European children and adolescents through the education and training of paediatricians and the practice of evidence-based medicine. Headquartered in Brussels, the EAP, through the European Board of Paediatrics (EBP), represents the “Paediatric Section” of the “European Union of Medical Specialists” (UEMS-SP), providing educational guidelines and training opportunities, as well as being involved in numerous initiatives related to the health and wellbeing of children and adolescents and research projects funded by the EU Commission.

The European Paediatric Association (EPA), formerly the “Union of National European Paediatric Societies and Associations” (UNEPSA, founded in 1976 by the presidents of national societies) is a forum for discussion of paediatric issues recognizing as a particular challenge the considerable differences in paediatric care among European countries. A major achievement was the initiation of clinical collaboration and research activities between the politically divided Eastern and Western European countries.

The European Confederation of Primary Care Paediatricians (ECPCP, founded in 2009) attributes more importance, value, and recognition to European primary-care paediatricians and their care of children. Their many accomplishments over the brief period of their existence include publications and statements on child health issues, as well as a comprehensive training program for aspiring primary care paediatricians.

One of the main objectives of this document is to raise awareness of the three paediatric societies to work together and create a unified and powerful voice across the European region that will effectively defend the health and rights of children and the profession that cares for them, as does the American Academy of Pediatrics in the 50 United States.

## Historical review

Since the founding of CESP (Confederation of European Specialists in Paediatrics) at a conference in Siena, Italy, in 1961, numerous dedicated European paediatricians have worked to advance the quality of care for European children.

Among the first issues addressed were:

- Paediatric education and training
- Interaction between paediatricians and general practitioners or other specialists
- Preventive paediatrics
- School medicine
- Children in the hospital
- Perinatal care
- Recognition and treatment of handicapped children

In order to agree on the definition of the scope of practice and specific competencies for paediatricians in Europe, paediatrics was defined as embracing “paediatrics and adolescent medicine” at the Athens meeting in 1986 and established as a medical specialty for human growth and development. After approval by the Union of European Medical Specialists (UEMS), despite objections from other sections, paediatrics “came of age” and was able to develop like internal medicine with structuring into subspecialties. In 1979 CESP was integrated as the Paediatric Section of the UEMS.

A European model for the training of paediatricians was developed by CESP representatives of the national professional paediatric organisations. The document “Paediatric Training in the European Community” (1) published in 1991, served as the basis for the founding of the “European Board of Paediatrics”.

Since the 1980s, working groups have been formed for specific areas of practice, starting with “paediatric training”, “accident prevention”, “harmonization of vaccination of children and adolescents in the EU” (2) and ethics (3–5). Contacts were established with other paediatric organizations such as the Union of National European Paediatric Societies and Associations (UNEPSA), the Association for Paediatric Education in Europe (APEE), the European Society for Paediatric Research (ESPR), the European Society for Social Paediatrics (ESSOP), the Club International de Pédiatrie Sociale, the Société Européenne de Recherche en Pédiatrie Ambulatoire (SERPA), but in particular with the scientific societies of the new paediatric specialties. In addition, exchanges with paediatric surgeons and child psychiatrists were promoted.

In 1993 J. RAMET became president of the European Board of Paediatrics and initiated training guidelines for primary, secondary, and tertiary care. He was successful in integrating the disciplines dealing with the paediatric systems into the structure of CESP and in achieving recognition by the UEMS of these, as paediatric subspecialties. As Secretary General from 1999, together with H. Helwig, he contributed to the further development of the relationship with the American Academy of Paediatrics (AAP) and in 2001 to changing the name of CESP to the “European Academy of Paediatrics (EAP)”.

In 2009 a European collaborative practice-based research network, EAP Research in Ambulatory Settings Network (EAPRASnet) was established. Among other educational activities the EAP has provided international meetings since 2006 where the EAP Congress & Master Course currently alternates with the meeting of the European Academy of Paediatric Societies (EAPS). In 2018 EAP opened its doors giving full constitutional rights to all countries of the European region. In 2019 EAP was recognized as the regional society for Europe and was given a seat in the standing committee of the International Paediatric Association (IPA). In November 2021 the annual EAP-EBP “European Board of Paediatrics Examination” was launched.

EAP collaborates closely with the major organizations related to children's health and development, like World Health Organization, UNICEF, European Medicine Agency, and European Center of Disease Control and Prevention. EAP is currently actively involved in several European EU Commission funded research projects supporting immunization (IMMUHUBS, RIVER EU, SEKI) and testing of high-risk medical devices (CORE-MD). During the last ten years more than sixty policy statements have been published on behalf of EAP.

In more than 60 years, as CESP/EAP developed into a major European medical organization, two other important societies were established (Table 1).

**Table 1:** Chronology and characteristics of paediatric organizations in Europe.

	Founded	Member countries (initial) **current**	Founding goals
Confederation of European Specialists in Paediatrics (CESP) *which later (2001) became known as* European Academy of Paediatrics (EAP)	1961	(6) **40**	To advocate, promote, and setting standards for attaining the highest quality of care for European children and adolescents through education of paediatricians and the practice of evidence-based medicine
Union of National European Paediatric Societies and Associations (UNEPSA), *which later (2007) became known as* European Paediatric Association (EPA)	1976	(18) **38**	Forum for discussion of paediatric issues recognizing as a particular challenge the considerable differences in paediatric care among European countries
European Confederation of Primary Care Paediatricians (ECPCP)	2009	**17**	To attribute more importance, value, and recognition to European primary-care paediatricians and their care of children.

The bold values mean the current number of member countries (as described in the headline).

The **Union of National European Paediatric Societies and Associations** (UNEPSA) was founded in Rotterdam in 1976 by the presidents of paediatric societies from 18 European countries. For the first 30 years of its existence, UNEPSA created a forum for discussion of paediatric issues with annual meetings, recognizing as a particular challenge the differences in paediatric care among European countries. During the “Cold War” years UNEPSA succeeded in overcoming the barriers between Eastern and Western European countries, thus initiating clinical collaboration and research activities across political boundaries that had previously been devoted primarily to determining the demographics of primary, secondary, and tertiary paediatrics in Europe. On the occasion of the renaming of UNEPSA to the **European Paediatric Association** (EPA, www.epa-unepsa.org/) in 2007, a review of these activities was published (6). EPA/UNEPSA currently has 38 member countries and organizes the biennial congress “Europaediatrics”.

The **European Confederation of Primary Care Paediatricians** (ECPCP, www.ecpcp.eu) was founded in 2009 to give more weight to European paediatricians in primary care. The ECPCP evolved from a precursor organization, SEPA/ESAP (Societé Européenne de Pédiatrie Ambulatoire), which was composed of individual paediatricians, whereas ECPCP, now also allows national professional associations to become members. Currently ECPCP is formed by 23 organizations from 17 nations in the WHO European Region.

Delegates meet once or twice a year and work together in five working groups: “Research”, “Prevention/Vaccination”, “Curriculum/Education”, “Environmental health”, and “Advocacy”. In addition to publications and statements on current child health issues, a “Curriculum in primary care paediatrics” has been developed and a training program for aspiring primary care paediatricians was published (7).

Although the initial focus of the ECPCP on the interests of primary care paediatricians led to their marginalization from other European paediatric societies, in recent years their collaborative approach on joint projects with other European paediatric societies has made them part of the European paediatric family.

In the following section the focus will be directed towards the characteristics of the EAP which represents the oldest European paediatric organization (Table 2).

**Table 2:** The individual areas of the EAP organizational structure, their functions, and current accomplishments.

Area	Function	Accomplishments
European board of paediatrics	Harmonize and maintain the highest possible paediatric training standards throughout Europe	(1) Elaboration of the UEMS European Training Requirements (ETRs) (2) Component of UEMS Multidisciplinary Joint Committees (3) Accreditation of Training Centres (4) Accreditation of CME events (5) Yearly European Board of Paediatrics Examination
Primary-care council	Address all somatic, psychological, and social aspects of prevention, diagnosis, treatment, and rehabilitation of newborns, infants, children, and adolescents in all outpatient settings	To help physicians in the care of children since in about 1/3 of the countries, children and adolescents are cared for exclusively by general practitioners whose level of training and experience in relation to this age group varies widely.
Secondary-/tertiary-care councils	Serve the scientific, educational, professional, and practice interests of hospital paediatrics and paediatricians dealing with paediatric sub-specialties	(1) Maintains the European Training Requirements (ETRs) of the current 14 recognized paediatric subspecialties. (2) Currently working to shape a common paediatric training approach, including paediatric basic training (“common trunk”).
Young-EAP (yEAP)	Network of young members that supports EAP's work in education, innovation, and advocacy.	(1) Collaboration on EAP projects. (2) Developing & working on own projects: (a) Migration & Health (b) Comparison of National training programs, and working and training conditions
European Academy of Paediatrics Research in Ambulatory Settings network (EPRASnet)	Composed of approximately 1,600 paediatricians from 43 countries working to enhance the quality of paediatric primary care through practice-based research.	Knowledge gained from studies influence changes in clinical guidelines and the adoption of policy statements of paediatric organizations and national governments
Strategic Advisory Groups (1) “Adolescent Health” (2) “Choosing Wisely” (3) “Complex Integrated Care” (4) “Ethics” (5) “Medicines for Children” (6) “Rare Diseases” (7) “Vaccination”	Develop ways to improve research, education, knowledge/skills, diagnostic and treatment recommendations as well as informed physician-patient communication.	(1) Peer-reviewed Publications (2) “Public Relations” carried out through regular press releases, newsletters, blogs, and campaigns using social media.

The listing of the SAGs is in alphabetical order and not in order of importance.

## European board of paediatrics (EBP)

The European Board of Paediatrics (EBP) is a standing committee and integral part of the EAP but also represents the executive body of the Paediatric Section of the Union for European Medical Specialists (UEMS-SP). As such, it must follow the statutes and rules of the UEMS, which is especially important when it comes to board votes and decisions.

The main role of the EBP is to harmonize and maintain the highest possible paediatric training standards throughout Europe. One of the main accomplishments of the Board has been the elaboration and regular update of the European Training Requirements (ETRs), which delineates the curriculum, assessment methodology for trainees, and requirements for trainers and training centres. The ETRs are developed jointly by the EBP and the European professional societies, adopted at a General Assembly of the EBP, and ultimately submitted to the Council of the UEMS for official recognition. The EBP's constructive collaboration and integration with the EAP and the paediatric specialty societies within the UEMS is key to improving the quality of education and patient care for children and adolescents across Europe.

The EBP is also responsible for the paediatric presence on the Multidisciplinary Joint Committees (MJCs) established by the UEMS to address training issues in emerging areas. As a UEMS section, the EBP has the same right as national bodies and specialist societies to request representation on MJCs. Other responsibilities of the EBP include the accreditation of training centres and of conferences for continuing medical education, as well as the coordination of specialty examinations.

Once a year, the EBP offers the EAP-EBP “European Board of Paediatrics Examination”, which was developed in cooperation with, and approved by, the Council for European Specialists Medical Assessments (CESMA) and UEMS with an online course (https://www.eapaediatrics.eu/core-knowledge-in-paediatrics/) offered prior to the exam. The test serves primarily as a knowledge-based examination for prospective paediatricians at the end of basic training, but also to assess the degree of knowledge at all levels of training as well as post-training.

## Primary-/secondary-/tertiary-care councils

The number of primary care paediatricians in Europe is decreasing. In about one third of the countries, children and adolescents are cared for exclusively by general practitioners whose level of training and experience in relation to this age group varies widely (8). The Primary–Care Council addresses all somatic, psychological, and social aspects of prevention, diagnosis, treatment, and rehabilitation of newborns, infants, children, and adolescents in outpatient settings such as public clinics, health centres, and public and private individual or group practices.

The Secondary–/Tertiary–Care Council serves the scientific, educational, professional, and practice interests of hospital paediatrics and paediatricians dealing with paediatric sub-specialties. The Council is responsible for maintaining the European training requirements of the 14 currently recognized paediatric subspecialties and is working to shape a common paediatric training approach, including paediatric basic training (“common trunk”).

Evidence-based training programs and continuous medical education are designed to ensure the highest possible quality of care for children and adolescents throughout Europe.

The Councils are represented on the European Board of Paediatrics (EBP) through relevant working groups and strategic advisory groups.

## Young EAP (yEAP)

Young EAP is a network of paediatric trainees who are members of national paediatric societies, currently from 25 European countries, founded in 2017 to support EAP's work in education, innovation, and advocacy. In addition to collaborating on various EAP projects, it has developed its own projects on migration & health, comparison of national training programs, and working and training conditions. Most recently, a network of European paediatric institutions was created to help children from Ukraine with complex medical conditions. The yEAP representatives have virtual meetings on a monthly basis to coordinate their agendas and generate new ideas as a “think tank”. Regular blogs on health (policy) issues are published on their website (https://www.eapaediatrics.eu/yeap/). The yEAP Chair is a voting member of the EAP Executive Committee.

## “Strategic advisory groups” (SAG)

The EAP Strategic Advisory Groups on “Adolescent Health”, “Choosing wisely”, “Complex Integrated Care”, “Ethics”, “Medicines for Children”, “Rare Diseases”, and “Vaccination” were established to develop ways to improve research, education, knowledge and skills, diagnostic and treatment recommendations for these areas of concern.

Members of all working groups meet twice a year at the EAP Winter and Spring Meeting to discuss current issues and develop important ideas and concepts to disseminate through publications. Public relations are carried out through regular press releases, newsletters, blogs, and campaigns using social media.

## SAG on adolescent health

In light of increasing health problems, chronic diseases, and potential long-term consequences of unhealthy lifestyles in adolescence, this strategic advisory group was established to develop educational initiatives and recommendations for youth health care. There is now a growing awareness that health professionals, particularly paediatricians and general practitioners, need to acquire specific knowledge and skills to communicate effectively with adolescents, in order to provide appropriate support and guidance.

## SAG on choosing wisely

The EAP has launched a “Choosing Wisely” working group, in line with the related international movement to promote communication between physicians and patients. The goal of this SAG is to target diagnosis and treatment, based on evidence, without duplicating tests or procedures already performed, thus minimizing potential harm to patients and costs to health care systems. Resources with recommendations from seven countries, paediatric initiatives, and useful links can be found on the EAP website.

The SAG on Choosing Wisely and the EAP Research in Ambulatory Settings network (EAPRASnet), in collaboration with the Japanese Paediatric Society, recently conducted a survey with a total of 3,353 records to identify the most common topics of overtesting and overtreatment in Europe and Japan (9).

## Complex integrated care task group

In 2021 the EAP established a multidisciplinary Working Group to reconcile the wide variation in terminology used to refer to children living with complex needs across clinical, research, and policy settings and to support the effective development of programs of care for this group of children and their families. A position paper examining the current definitions of complex care existing in the literature concluded the phrase “complex and integrated care” to be the most adequate (10). Currently a survey is ongoing to support service development in complex care.

## SAG on ethics

Since the establishment of the Strategic Advisory Group on Ethics in 1997, more than 30 papers on various topics have been published (3–5) and statements on critical issues have been addressed to the ministries of health of EU countries. Workshops have been organized to recruit members and share their different opinions on “hot issues and topics” within paediatric ethics. The positive broadly networked working atmosphere has already resulted in three multinational research projects funded under EU programs.

## SAG on medicines for children

The goal of the Strategic Advisory Group on Medicines for Children is to obtain access to safe, modern, and affordable medicines that ensure diagnosis, treatment, and prevention of diseases for all children and adolescents in Europe.

Urgent issues include appropriate formulations and dosing, correct administration of medicines, lack of medicines suitable for children, excessive prices of some medicines for rare diseases, lack of investment in medicines for specific paediatric indications/needs, and insufficient infrastructure/networks to conduct multicentre paediatric studies in Europe resulting in frequent prescriptions of off-label medicines.

## SAG on rare diseases

A 2009 report of the European Organization of Rare Disease Patients (EURORIDS), (“Voice of 12,000 Patients”), emphasized, among other things, problems that went from delayed diagnoses to the actual rejection of health professionals to care for these children because of the complexity of the associated problems. Most of the more than 6,000 rare diseases defined as “life-threatening or chronically debilitating diseases with low prevalence and high complexity” occur in childhood. Affected patients, their families, and caring physicians require a high level of support (11).

Adequate early detection, confirmation of diagnosis, and long-term treatment and follow-up are prerequisites that help guarantee the best possible health for children with rare and chronic diseases. The SAG on Rare Diseases supports actions to globally strengthen a comprehensive approach for every child with a rare, chronic disease.

## SAG on vaccination

The EAP Vaccination Strategic Advisory Group (VSAG) forms an interactive network of delegates closely related to their respective national vaccination programs.

Optimization of vaccination coverage rates by overcoming increasing vaccination scepticism, the introduction of electronic vaccination records, sufficient vaccine supply, and harmonization of vaccination programs within the European Region are the main goals of the advisory group. Collaborations with IPA, WHO/UNICEF, the European Commission (Coalition for Vaccination, HaDEA), ECDC, European Society of Paediatric Infectious Diseases (ESPID), European Scientific Working group on Influenza (ESWI), VACCELERATE and other international organizations support these efforts.

The EAP VSAG is involved in several EU projects to improve immunization rates, especially in disadvantaged and hard-to-reach populations (ImmuHubs, RIVER EU) and is co-founder of the platform for Standardized Education and Knowledge on Immunization (www.seki.eu).

Publications, statements, and blogs have been produced in collaboration with EAPRASnet, the Ethics SAG, ESPID, and Young EAP (12–14). Members of the SAG regularly contribute to international congresses, symposia, and webinars, and also act as advisors to international and national health authorities.

## EAPRASnet

EAPRASnet (European Academy of Paediatrics Research in Ambulatory Settings network) was established in 2009 to enhance the quality of paediatric primary care through practice-based research. A “steering group” of research advisors forms a research network (info@eaprasnet.org) with paediatric offices in European and Mediterranean countries, and cooperates with its counterpart in the USA, the “Pediatric Research in Office Settings” (PROS) which is the practice-based research network of the American Academy of Pediatrics (AAP).

EAPRASnet acts as a “research laboratory,” so to speak, for paediatric primary care, with approximately 1,600 active paediatricians from 43 countries. Data on research questions collected through surveys are therefore available relatively quickly and are statistically meaningful. High response rates are achieved with the help of national coordinators.

In the course of the initial recruitment survey for participation to this network in 2010, four priority research areas were identified: indicators of quality of care, communication with parents, obesity, and ADHD. Interest in the topics and improving quality of care were primary incentives; however, lack of time was the most frequently cited barrier to participation (15). Currently 12 published surveys with increasing participation on different topics are listed on the EAP homepage (https://www.eapaediatrics.eu/eaprasnet/).

In 2018, EAPRASnet entered a new phase of its short history by investigating, for the first time, factors influencing parental vaccination decisions based on their views toward vaccinating their children by surveying (approximately 6,000) parents in paediatric and general medical offices in 18 European countries (13).

The goal of EAPRASnet is to have new knowledge gained from their studies influence and change clinical guidelines or policy statements of paediatric organizations, just as the results from the studies conducted by AAP-PROS have already positively influenced decisions in the US health care system.

## Perspectives

The aspiration to merge the paediatric organizations of Europe into a common structure in order to be able to advocate for child and adolescent health in Europe and internationally with one strong voice, analogous to the AAP, was the trigger for a “summit” of the three largest paediatric organizations (EAP, ECPCP, and EPA) in Vienna in December 2017 (16). In the meantime, mutual invitations, joint events, projects, and publications (17) have indicated an increasing coming together. Continuous efforts of great personalities, putting individual interests aside, are a prerequisite for achieving the high goal of a common representation of interests for child and adolescent health in Europe. With the child at the centre of our attention, we need to look for solutions not conflicts!

## Conclusion

Promoting the health and well-being of children and adolescents in Europe through education, research, and advocacy is a high ethical goal. This requires sound educational guidelines, a well-coordinated platform for knowledge and exchange of ideas, collaboration on research and clinical projects, and comprehensive continuing education.

The European Academy of Paediatrics (EAP) and the European Board of Paediatrics (EBP) as the paediatric section of the European Union of Medical Specialists (UEMS PS) fulfil all these requirements. Other organizations such as the European Paediatric Association (EPA) and the European Confederation of Primary Care Paediatricians (ECPCP) contribute substantially to these areas.

Constructive collaboration among European paediatric organizations in the sense of a single, strong, unifying voice is an important and essential step in maintaining and promoting the best possible care for child and adolescent health in Europe.
